# Successful Biological Invasion despite a Severe Genetic Load

**DOI:** 10.1371/journal.pone.0000868

**Published:** 2007-09-12

**Authors:** Amro Zayed, Şerban A. Constantin, Laurence Packer

**Affiliations:** Department of Biology, York University, Toronto, Ontario, Canada; University of Sheffield, United Kingdom

## Abstract

Understanding the factors that influence the success of ecologically and economically damaging biological invasions is of prime importance. Recent studies have shown that invasive populations typically exhibit minimal, if any, reductions in genetic diversity, suggesting that large founding populations and/or multiple introductions are required for the success of biological invasions, consistent with predictions of the propagule pressure hypothesis. Through population genetic analysis of neutral microsatellite markers and a gene experiencing balancing selection, we demonstrate that the solitary bee *Lasioglossum leucozonium* experienced a single and severe bottleneck during its introduction from Europe. Paradoxically, the success of *L. leucozonium* in its introduced range occurred despite the severe genetic load caused by single-locus complementary sex-determination that still turns 30% of female-destined eggs into sterile diploid males, thereby substantially limiting the growth potential of the introduced population. Using stochastic modeling, we show that *L. leucozonium* invaded North America through the introduction of a very small number of propagules, most likely a singly-mated female. Our results suggest that chance events and ecological traits of invaders are more important than propagule pressure in determining invasion success, and that the vigilance required to prevent invasions may be considerably greater than has been previously considered.

## Introduction

Invasive species are a serious threat to biodiversity conservation [1]–[3] and the global economy [4], and understanding the patterns and processes governing the establishment and spread of exotic organisms is of primary importance. The success of invasive species poses a paradox [5], [6]: How can founder populations, expected to have reduced genetic diversity and thereby reduced fitness and adaptability, colonize and dominate large areas of new habitat? Both theoretical and empirical studies suggest that the number of introduced exotic propagules is often positively correlated with invasion success [6]–[9], a concept referred to as propagule pressure. Recent genetic studies have shown that invasive populations typically exhibit minimal, if any, reductions in genetic diversity [10]–[13], suggesting that large founding populations and/or multiple introductions are required for the success of biological invasions [8]–[11], consistent with predictions of the propagule pressure hypothesis.

*Lasioglossum leucozonium* (Schrank) (Hymenoptera: Halictidae) is a solitary ground-nesting bee which has a single generation per year, and overwinters underground as singly-mated females [14]. Biogeographic and phylogenetic evidence show that *L. leucozonium* was introduced to North America (NA) from Europe [15] and museum collections suggest that this occurred at least a century ago [14]. Until recently, this bee was commonly considered native to NA [15], where it can be found in large numbers throughout its range [16], [17]. We studied the population genetics of *L. leucozonium* by quantifying genetic variation at six microsatellite loci [18] in 254 females from 12 NA populations, and one French population expected to be typical of the introduction's source (Table S1). In addition, using bees sampled from excavated nests, we quantified levels of allelic richness at the usually hypervariable hymenopteran sex-determination locus [19] in NA. Here we demonstrate that *L. leucozonium* experienced a severe bottleneck, and consequently a heavy genetic load, during its introduction to NA. Further, modeling analyses suggest that the founder population consisted of a very small number of propagules, most likely a singly-mated female.

## Results

Introduced populations of *L. leucozonium* had very low levels of genetic diversity. Genetic variation at the microsatellite loci was significantly reduced in NA when compared to the French population (Table 1; Wilcoxon rank-sum test, *N* = 6, *p* = 0.0039 for both allelic richness and expected heterozygosity). We did not observe more than 3 alleles per locus in NA, and average allelic richness was reduced by 76% when compared to the French population (Table 1), a higher reduction in allelic richness than found in any other introduced animal surveyed for that parameter [10]. Given that we sampled 13 times more individuals in NA than in Europe, and from a wider geographic range, our analysis underestimates genetic diversity in the immigrants' region of origin [20], and thus the observed reductions in genetic diversity in NA are likely underestimates of the true values.

**Table 1:** Expected heterozygosity, *H*_exp_, and average allelic richness, *N*_A_ in native and introduced *L. leucozonium* populations.

Locus	*H*_exp_		*N*_A_ *	
	Native	Introduced	Native	Introduced
Leu-A22	0.842	0.477	13.00	2.00
Leu-A52	0.806	0.654	9.00	3.00
Leu-A73	0.796	0.534	8.00	2.74
Leu-B34	0.863	0.307	10.00	2.00
Leu-B60	0.951	0.558	18.00	2.91
Leu-B72	0.827	0.607	7.00	2.99
***Average***	***0.848***	***0.523***	***10.83***	***2.61***

* Based on a corrected sample size of 18 females.

After a population bottleneck, allelic richness is reduced at a higher rate than is heterozygosity and this results in an apparent excess in heterozygosity over that expected given the observed allelic richness and assuming a stable population at mutation-drift equilibrium [21]. All NA populations significantly exhibited such an excess in heterozygosity (*p*<0.05 for all tests), while the French population did not (*p* = 0.50). Given that the signature of a bottleneck is detectable only within a short time period after the event [21], and assuming reasonable effective population sizes for bees (see methods), we estimate that *L. leucozonium* was introduced to NA between 50 to 500 years ago. This period encompasses the 18^th^ and 19^th^ centuries when many ground-associated insects were introduced to NA through soil-ballast associated with transatlantic shipping [22].

North American populations of *L. leucozonium* did not exhibit any genetic structure (Global *F*_ST_ = 0.003-not significantly different from zero, *p* = 0.33). All pairwise estimates of genetic differentiation were not significantly different from zero (pairwise *F*_ST ,_ *p*>0.05 for all tests), even between populations separated by more than 1,400 Km. This is highly uncharacteristic of native bee populations which, due to central-place foraging and nest-building, often show significant levels of population genetic structure [23]–[26]. Lack of genetic differentiation and isolation by distance are most likely due to historical artifacts (i.e. recent common ancestry), rather than current ongoing gene flow, especially given the distances separating the studied populations.

In addition to the microsatellite dataset, we also sequenced a 618 bp fragment of the mitochondrial gene cytochrome *c* oxidase I (COI) in 40 NA and 18 French bees (Genbank EF629474–EF629531). All sequenced bees, from both regions, shared the same COI haplotype supporting the view that *L. leucozonium* is invasive in NA [15]. Unfortunately, lack of variation at COI renders the mtDNA dataset uninformative for population genetic analysis.

In bees, and many other Hymenoptera, sex is determined by the complementary actions of alleles at a single autosomal locus [19], [27], [28]: Heterozygotes at that locus are female, while hemizygotes and homozygotes are haploid and effectively sterile diploid males respectively. By genotyping sexed pupae from excavated *L. leucozonium* nests in NA, we found 29.7% of diploids to be male. This yields an estimate of 3.36 (+/−1.06 SD) effective sex-determination alleles in NA, the lowest value recorded for any exotic hymenopteran [29]. Since the sex-determination locus experiences strong balancing selection and negative frequency dependent selection, natural hymenopteran populations usually maintain 9 to 20 sex-determining alleles, with some populations reportedly having>40 alleles [27], [30].

**Figure 1:**
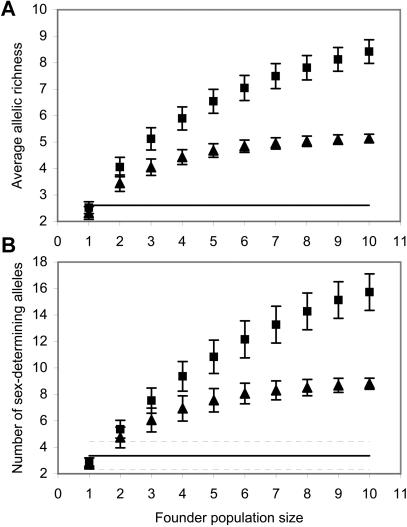
The allelic richness observed in introduced *L. leucozonium* populations, at both microsatellite loci and the sex-determination locus, is best explained by the introduction of a single founder. (A) The allelic richness observed in NA (solid line), and that estimated for founder populations derived from the French population (squares) or a source population with 50% less allelic richness (triangles). (B) The observed number of sex-determination alleles in NA (solid line) and those estimated for founder populations derived from a source population with 20 (squares) or 9 (triangles) sex-determining alleles–the range expected in natural hymenopteran populations. Dashed lines and error bars indicate standard deviation.

To explore the size of the founding population, we assumed a simple demographic scenario where the introduced propagules founded a population that immediately started to increase. Therefore, barring mutation, the current level of allelic richness in NA populations is reflective of that of the founder population (refer to Methods). We can therefore estimate the number of founders which colonized NA by comparing the allelic richness at microsatellite loci observed in the introduced population to that derived by randomly sub-sampling different numbers of individuals from the French sample which is expected to be typical of the source population (refer to Methods). Given the observed reductions in allelic richness in NA when compared to France, the introduced population was most likely founded by one singly-mated female (Figure 1A). Even a source population with 50% less allelic richness than the French sample–a value that is much higher than the observed standard deviation of that parameter in natural solitary bee populations (refer to Methods)–still yields one singly-mated female as the most likely founder population size (Figure 1A). Similarly, assuming a source population with 9 or 20 sex-determining alleles, the range expected of natural hymenopteran populations [27], and since females must be heterozygous at the sex-determination locus, a founder population of more than one female would almost certainly have carried more alleles than are now observed in NA (Figure 1B). Both of the estimates are consistent with the observation that no more than 3 alleles were observed at any microsatellite locus in NA (Table 1).

**Figure 2:**
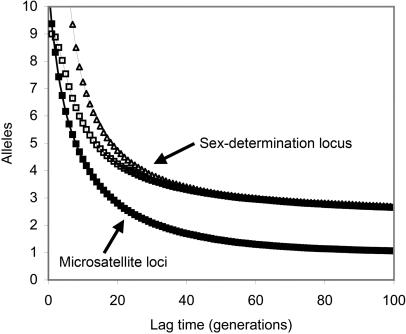
Lack of concordance in allelic richness between microsatellite loci and the sex-determination locus rule out a ‘lagged’ invasion scenario. In simulated founder populations of 100 bees, average allelic richness at the microsatellite loci (black squares) declined at a faster rate during the lag period when compared to the sex-determination locus initialized with either 9 (open squares) or 20 (open triangles) alleles. Average allelic richness at the microsatellite loci was transiently concordant only with that of the sex-determination locus at 9 alleles-much higher than observed in the introduced *L. leucozonium* population where both types of loci had ∼3 alleles.

An alternative invasion scenario can be envisioned: *L. leucozonium* could have been introduced to NA via a larger population which experienced a lag period of no population growth, increasing the effects of drift, followed by subsequent growth and spread. Anecdotal evidence for lagged invasions exists, although recent experimental studies suggest that lags are likely caused by our reduced ability to detect small introduced populations, rather than actual periods of no population growth [31]. Further, lack of population structure, isolation by distance, and differences in genetic diversity between *L. leucozonium* populations in NA suggest that drift was insignificant post the introduction [12], [32]–[37]. Nevertheless, if *L. leucozonium* experienced a period of no growth upon its introduction in NA, then we would expect drift to have reduced genetic variation at neutral microsatellite loci [38] at a faster rate than at the sex-determination locus due to strong balancing selection acting on the latter [30], [39]. This implies that the observed concordance in allelic richness at microsatellite loci and the sex determination locus (∼3 alleles each) in NA populations of *L. leucozonium* would occur rarely, or not at all, if this species was founded by a larger population which experienced a lag period. We explored this alternative demographic scenario by simulating a founding population (N = 100 bees) which experienced a lag period prior to population expansion. The concordance of allelic richness between the two types of loci occurred only transiently and at much higher values (∼9 alleles) than observed in our dataset (Figure 2), rendering this demographic scenario unlikely.

## Discussion

Taken together, highly reduced levels of genetic variation in introduced *L. leucozonium* populations along with significant bottleneck tests and lack of population structure suggest that this bee experienced a single and severe founder event during its introduction to North America. Multiple founding events, or a large founding population, would have acted to increase genetic diversity and/or increase genetic differentiation in the introduced range [10]–[12]. To explore the size of the founding population, we modelled the allelic richness that would be expected of introduced populations using what we believe to be the two most parsimonious invasion scenarios: 1) A number of founders are introduced which establish a population that immediately starts to increase in size. 2) A number of founders are introduced experiencing a lag period of no population growth prior to population increase and expansion. Lack of concordance between allelic richness at the sex-determination locus and microsatellite loci renders a ‘lagged’ invasion scenario unlikely. Under the first invasion scenario, only the introduction of a singly-mated female is consistent with the observed levels of allelic richness in NA–a finding that is robust to the expected deviations of allelic richness in the hypothetical source population. We are thus confident in the estimate of one singly-mated female as the most likely founder size, although we acknowledge that we did not model other possible but less parsimonious invasion scenarios which may lead to higher estimates.

Although our analyses show that *L. leucozonium* was introduced to NA via a very small number of propagules, the genetic data also suggest that the bee's subsequent spread in NA involved a larger number of individuals. If *L. leucozonium* spread across NA via additional small founder events, creating serial bottlenecks, we would expect to see a much stronger signature of genetic drift in our dataset, such as significant population structure, isolation by distance, and reductions of genetic diversity from the initial introduction site (the east coast) to the colonizing front [32], [37]; these patterns are absent. Although current gene flow across *L. leucozonium*'s North American distribution can obliterate the signature of a serial founder event, we feel that such a scenario is unlikely given the distances involved as well as the lack of evidence for long distance dispersal in bees [24].

In isolated haplodiploid populations with complementary sex determination, reductions in the number of sex-determination alleles increase the production of inviable or effectively sterile diploid males, which in turn reduce population growth rates and effective sizes potentially creating a rapid extinction vortex [40], [41]. As a result, introductions of solitary bees are unlikely to become established because diploid male production imposes the largest genetically-induced threat to population viability [40], [42]. Paradoxically, *L. leucozonium* managed to invade NA despite this enormous genetic load. Although several species of ants and wasps have become highly invasive in their introduced range, none have experienced reductions in genetic diversity on the same scale as *L. leucozonium* [12], [29], [43], [44]. Further, *L. leucozonium* is solitary, and thus lacks the behavioural repertoires implicated in the success of invasive social hymenopterans [45].

However, *L. leucozonium* exhibits several ecological traits which may have aided in its establishment and spread in NA. *L. leucozonium* is polylectic [14] and diet generalization has been suggested to play a role in the success of invasive species [46], [47]. It is interesting to note that *L. leucozonium* is associated with several exotic weeds such as *Chicorium* and *Hieracium* [14], [15], which may have facilitated the bee's establishment and spread in NA. Further, nests of *L. leucozonium* in NA have low rates of parasitism compared with closely related species studied in the same habitats [16], which may be attributed to unusual nest building and guarding behaviors [16], [48]. Finally, females of *L. leucozonium* have an above-average productivity for solitary bees (Constantin et al, unpublished), partially counteracting the fitness reductions imposed by diploid male production [40]. Nevertheless, assuming modest carrying capacities (K>100) and the observed productivity data for this bee in NA, stochastic modeling indicates that both extinction and persistence of a population founded by a single immigrant were likely events (Figure S1A), stressing the important but often neglected role of chance in biological invasions [49], [50]. In simulations with K>100 bees, the risk of extinction per generation for a population founded by one mated female declined rapidly over time, such that populations which managed to survive and increase in size during the first two generations had a negligible risk of extinction (Figure S1B), echoing the observations of several experimental studies [31], [51].

Our study provides evidence of a successful biological invasion despite a severe founder event and genetic load. Moreover, the introduced population of *L. leucozonium* managed to spread and persist in NA for over a century despite a ∼30% reduction in its population growth potential. The literature on intentional insect introductions for biological control provides several examples of successful establishment from a few (<10) individuals [47], [52]. For example, several species of parthenogenic parasitoid wasps in the Encyrtidae were reared in the laboratory from 1 or 2 females, and the resulting populations were released and successfully established in the field [52]. More impressively, a single gravid female of the chrysomelid beetle *Galerucella calmariensis*, introduced to control the invasive weed *Lythrum salicaria*, established a field population which persisted for at least 5 years [51]. Similarly, a single female of the psyllid bug, *Arytainilla spartiophila*, introduced to control the broom *Cytisus scoparius*, established a field population which persisted for a least 3 years [31]. Therefore, successful invasion from a very small number of propagules may be more common than previously realized.

Although propagule size and population persistence are usually positively correlated, as expected by theory and demonstrated through empirical studies [6]–[9], it is also possible for persistence to be nearly independent of propagule size [31], [51], especially when density-independent factors (e.g. habitat size, conditions) heavily influence population persistence and/or when introduced animals have high growth rates [5], [51]. Our findings therefore suggest that, in determining invasion success, propagule pressure may be of secondary importance to chance events, the ecological traits of exotic species, and the properties of the invaded ecosystem. Finally, if we are to achieve success in reducing the incidence of biological invasions, much more vigilance will be needed to reduce the unintentional introduction of exotic organisms–more so than has been previously considered.

## Materials and Methods

### Sampling, DNA isolation, and PCR

Females were collected as they foraged on flowers (refer to Table S1 for sampling locations and sizes). In addition to the collected bees, we also marked 100 nests at site LL6 in early June 2006, and excavated 32 in mid July 2006. Pupae from excavated nests were sexed based on differences in morphology. We extracted genomic DNA from the thorax of adults/pupae using a DNeasy Tissue Kit (QIAGEN) and genotyped the bees at the following microsatellite loci: Leu-A22, Leu-A52, Leu-A73, Leu-B34, Leu-B60, and Leu-B72 following standard protocols [18]. We sequenced a 618 bp fragment of COI in 40 NA and 18 French bees following standard protocols [53] at the Canadian Centre for DNA Barcoding (Guelph, Ontario, Canada).

### Population genetic analyses

We used SEQUENCHER (Gene Codes Corp.) to align the COI sequences and obtain a consensus sequence and manually determined the number of haplotypes based on sequence differences. No deviations from Hardy-Weinberg and linkage equilibrium were observed in the microsatellite dataset, as tested in FSTAT [54], using 15,000 randomizations. We estimated expected heterozygosity, allelic richness and both global and pairwise genetic differentiation (*F*_ST_) using FSTAT. The significance of *F*_ST_ estimates was determined using 15,000 randomizations of genotypes among samples [54]. When appropriate, we corrected for multiplicity of statistical tests using standard procedures [54], [55]. We used the non-parametric Wilcoxon rank-sum test [56] to statistically test the null hypothesis of no difference in mean heterozygosity and allelic richness between NA and the French population.

We used the software BOTTLENECK [21] to compare deviations from the observed heterozygosity at each locus in each *L. leucozonium* population with that expected at mutation-drift equilibrium. We ran our tests using both the infinite allele model (IAM), and the two-phased model (TPM) of mutation since very few microsatellite loci strictly follow the stepwise mutation model [21]. The results of both IAM and TPM models were consistent, and we report 1-tailed *p* values for the TPM tests only. The signature of a bottleneck is detectable within 0.5*N*_e_-5*N*_e_ generations after the event [21], where *N*_e_ is the effective population size. We estimated the time of the bottleneck assuming *N*_e_ = 100, representing the higher end of empirical estimates for bees [41].

We used maximum likelihood [57] to estimate the frequency of diploids that are male and the effective number of sex-determination alleles from *L. leucozonium*'s nest data. Since this method assumes that sampled nests contribute an equal number of progeny, we developed software to randomly sample a single diploid individual from each nest containing a sexed pupa, before applying the maximum likelihood equations to estimate the parameters of interest. This process was repeated 1000 times. Loci under strong balancing selection, such as the complementary sex-determination gene, show lower values of genetic differentiation when compared to neutral loci [39]. Given the lack of genetic structure between *L. leucozonium* populations in NA at neutral microsatellite loci, we therefore expect that our estimates of diploid male production from LL6 are representative for all of NA. In the analysis above, we assume that *L. leucozonium* has single-locus complementary sex determination (sl-CSD), the ancestral sex determining mechanism in the Hymenoptera [27], [28]. This assumption is strongly supported since high levels of diploid male production, as seen in our dataset, can only be explained by sl-CSD as none CSD systems do not produce diploid males, and multiple-locus CSD acts to reduce frequencies of diploid male production over sl-CSD expectations [27], [28]. Furthermore, no bee species has been shown to have a sex determination system other than sl-CSD [19], [27], [28]. Finally, *L. leucozonium* nests with multiple sexed diploid progeny containing diploid males (i.e. matched mated nests) had an average frequency of diploids that are male = 43% (SD = 15%), which is not significantly different from the 50% prediction of sl-CSD. Assuming the least variable form of multiple-locus CSD (two loci each with two alleles), a matched mated nest should have an average diploid male frequency of only 25%, much lower than observed. Therefore, both the number of diploid males produced and their ∼50% frequency in matched-mated nests are consistent with sl-CSD as the only plausible system of sex-determination in *L. leucozonium*.

### Founder population size

Previously published *L. leucozonium* mtDNA sequences from France and NA are nearly identical [58] and all 40 COI sequences obtained for this study perfectly matched the previously published French haplotype as well as the haplotype found in our 18 French bees, supporting the view that our French sample is typical of the introduction's source population. Our data (Constantin et al., unpublished) show that *L. leucozonium* females singly-mate, the norm for bees [59], hence any possible founder (i.e. singly-mated female) can have at most three alleles at each locus. To explore the size of the founding population, we randomly resampled 3N alleles at each locus, where N is the size of the founder population in units of singly-inseminated females, from a list of alleles and their counts in the French sample. Sampling was conducted without replacement for 1000 iterations for values of N = 1 to 10. We then estimated the average allelic richness in the simulated founder populations, and compared it to that observed in the pooled NA sample. Similarly, we also randomly re-sampled without replacement 3N sex-determining alleles for N = 1 to 10 founders from a hypothetical population containing 9 or 20 sex-determining alleles at equilibrium frequency–the range expected in natural hymenopteran populations [27]. Since females must be heterozygous at the sex locus, our sampling program ensured that each founder had 2 different sex alleles, representing a female's heterozygous genotype, as well as an additional allele obtained through mating, which can match any of the first two alleles.

In estimating the founder population size using the method outlined above, we assume that the founding population was either derived from the French sample, or a population with a similar level of average allelic richness (i.e. allelic identity is not taken into account). Average allelic richness does not greatly vary between populations of solitary bees in their natural range: the standard deviation in that parameter derived from microsatellite studies sampling 5 or more populations averaged only 15.21% of the mean [23], [26], [60], [61]. To investigate the robustness of our estimate of founder population size to changes in the average allelic richness of the source population, we repeated the analysis assuming that the source population had a 50% reduction in allelic richness compared to France. We achieved this by randomly deleting half of the alleles at each locus from the French dataset, then randomly allocating the deleted allele counts over the surviving alleles. We then randomly sampled 3N alleles in the same manner as described above for N = 1 to 10.

We also assume that the founding population immediately increased in size once introduced to NA (i.e. allelic richness in the founding population was not reduced by drift, and is thus reflective of present day richness in NA, see Figure S2). We used stochastic modelling [40] to explore an alternative scenario where *L. leucozonium* invaded NA via a larger population which experienced a lag period prior to spread, by simulating a founding population with N = 100 bees which was kept constant in size for a lag period of up to 100 generations. We assumed that *L. leucozonium* females produce an average of 8.35 eggs over their lifetime with a 1∶1 (haploid:diploid) sex ratio , as observed from our nest samples (Constantin et al., unpublished). We simulated 6 neutral loci, each starting with same number of alleles, and their frequencies, as found in the French sample, in addition to simulating the sex-determination locus initially with 9 alleles or 20 alleles at equilibrium frequency. The simulations were repeated for 1000 iterations, and we monitored how drift reduced the allelic richness in extant populations at the simulated microsatellite loci and the sex-determination locus during the lag period. Similar simulations with smaller founder sizes (<50 bees) were conducted but all such populations went extinct during the lag period (Data not shown) due to the diploid male extinction vortex [40].

### Stochastic modelling

We examined the extinction dynamics of a haplodiploid population founded by one singly-mated female using stochastic models [40], assuming the observed productivity and sex ratio estimates derived from our nest data (Constantin et al., unpublished), and assuming that the single founder was not ‘matched-mated’ (i.e. her and her mate do not share a sex-determination allele in common). The stochastic simulations were conducted for K = 50 to 1000, assuming diploid males are effectively sterile. The simulations were projected forward for 100 generations and repeated for 1000 iterations. For each value of K, we repeated the simulations 5 times to estimate the standard deviation of the probability of extinction.

## Supporting Information

Table S1 Sample sizes and locations for *L. leucozonium*
(0.04 MB DOC)

Figure S1 Chance and the establishment of the introduced population. (A) The probability of extinction, P(E), for a population established by one singly-mated female reached an asymptote with increasing K. Both extinction and persistence of the founder population were likely. Error bars indicate standard deviation. (B) Populations which survived the first two generations after the founding event had a negligible risk of extinction (K = 1000 bees).
(5.10 MB TIF)

Figure S2 Allelic richness is maintained in a small but initially expanding founder population. The graph was generated by modeling a population with K = 5000, founded by one-singly mated female with 3 microsatellite alleles (black line) and 3 sex-determination alleles (grey line). Average allelic richness and average population size (black triangles) of extant populations are plotted on the left and right y-axis respectively. Mutation was not simulated.
(3.66 MB TIF)

## References

[1] Clavero M, Garcia-Berthou E (2005). Invasive species are a leading cause of animal extinction.. Trends Ecol Evol.

[2] Gurevitch J, Padilla DK (2004). Are invasive species a major cause of extinctions?. Trends Ecol Evol.

[3] Simberloff D, Von Holle B (1999). Positive interactions of nonindigenous species: invasional meltdown?. Biol Invasions.

[4] Pimentel D, Lach L, Zuniga R, Morrison D (2000). Environmental and economic costs of nonindigenous species in the United States.. BioScience.

[5] Frankham R (2005). Resolving the genetic paradox in invasive species.. Heredity.

[6] Allendorf FW, Lundquist LL (2003). Introduction: Population biology, evolution, and control of invasive species.. Conserv Biol.

[7] Kolar CS, Lodge DM (2001). Progress in invasion biology: predicting invaders.. Trends Ecol Evol.

[8] Lockwood JL, Cassey P, Blackburn T (2005). The role of propagule pressure in explaining species invasions.. Trends Ecol Evol.

[9] Colautti RI, Grigorovich IA, MacIsaac HJ (2006). Propagule pressure: A null model for biological invasions.. Biol Invasions.

[10] Wares JP, Hughes AR, Grosberg RK, Sax DF, Stachowicz JJ, Gaines SD (2005). Mechanisms that drive evolutionary change: insights from species introductions and invasions.. Species Invasions: Insights into Ecology, Evolution, and Biogeography.

[11] Kolbe JJ, Glor RE, Rodriguez Schettino L, Lara AC, Larson A (2004). Genetic variation increases during biological invasion by a Cuban lizard.. Nature.

[12] Johnson RN, Starks PT (2004). A surprising level of genetic diversity in an invasive wasp: *Polistes dominulus* in the northeastern United States.. Ann Entomol Soc Am.

[13] Lavergne S, Molofsky J (2007). Increased genetic variation and evolutionary potential drive the success of an invasive grass.. Proc Natl Acad Sci USA.

[14] McGinley RJ (1986). Studies of Halictinae (Apoidea: Halictidae), I: Revision of New World *Lasioglossum* Curtis.. Smithson Contrib Zool.

[15] Giles V, Ascher JS (2006). A survey of the bees of the Black Rock Forest preserve, New York (Hymenoptera: Apoidea).. J Hym Res.

[16] Atwood CE (1933). Studies on the Apoidea of western Nova Scotia with special reference to visitors to apple bloom.. Can J Res.

[17] Grixti JC, Packer L (2006). Changes in the bee fauna (Hymenoptera: Apoidea) of an old field site in southern Ontario, revisited after 34 years.. Canadian Entomologist.

[18] Zayed A (2006). Characterization of microsatellite loci from the solitary sweat bees *Lasioglossum leucozonium* and *Lasioglossum oenotherae* (Hymenoptera, Halictidae).. Mol Ecol Notes.

[19] Beye M, Hasselmann M, Fondrk MK, Page RE, Omholt SW (2003). The gene csd is the primary signal for sexual development in the honey bee and encodes a new SR-type protein.. Cell.

[20] Petit RJ, Mousadik AE, Pons O (1998). Identifying populations for conservation on the basis of genetic markers.. Conserv Biol.

[21] Cornuet JM, Luikart G (1996). Description and power analysis of two tests for detecting recent population bottlenecks from allele frequency data.. Genetics.

[22] Lindroth CH (1957). The Faunal Connections Between Europe and North America..

[23] Zayed A, Packer L (2007). The population genetics of a solitary oligolectic sweat bee, *Lasioglossum* (*Sphecodogastra*) *oenotherae* (Hymenoptera: Halictidae)..

[24] Packer L, Owen R (2001). Population genetic aspects of pollinator decline.. Conserv Ecol.

[25] Zayed A, Packer L, Grixti JC, Ruz L, Toro H (2005). Increased genetic differentiation in a specialist versus a generalist bee: implications for conservation.. Conserv Genet.

[26] Danforth BN, Ji S, Ballard LJ (2003). Gene flow and population structure in an oligolectic desert bee, *Macrotera* (*Macroteropsis*) *portalis* (Hymenoptera: Andrenidae).. J Kans Entomol Soc.

[27] Cook JM, Crozier RH (1995). Sex determination and population biology of the Hymenoptera.. Trends Ecol Evol.

[28] van Wilgenburg E, Driessen G, Beukeboom L (2006). Single locus complementary sex determination in Hymenoptera: an “unintelligent” design?. Front Zool.

[29] Ross KG, Vargo EL, Keller L, Trager JC (1993). Effect of a Founder Event on Variation in the Genetic Sex-Determining System of the Fire Ant *Solenopsis invicta*.. Genetics.

[30] Yokoyama S, Nei M (1979). Population dynamics of sex-determining alleles in honey bees and self-incompatibility alleles in plants.. Genetics.

[31] Memmott J, Craze PG, Harman HM, Syrett P, Fowler SV (2005). The effect of propagule size on the invasion of an alien insect.. J Anim Ecol.

[32] Hutchison DW, Templeton AR (1999). Correlation of pairwise genetic and geographic distance measures: Inferring the relative influences of gene flow and drift on the distribution of genetic variability.. Evolution.

[33] Goodisman MAD, Matthews RW, Crozier RH (2001). Hierarchical genetic structure of the introduced wasp *Vespula germanica* in Australia.. Mol Ecol.

[34] Tsutsui ND, Case TJ (2001). Population genetics and colony structure of the Argentine ant (*Linepithema humile*) in its native and introduce ranges.. Evolution.

[35] Buczkowski G, Vargo EL, Silverman J (2004). The diminutive supercolony: the Argentine ants of the southeastern United States.. Mol Ecol.

[36] Henshaw MT, Kunzmann N, Vanderwoude C, Sanetra M, Crozier RH (2005). Population genetics and history of the introduced fire ant, *Solenopsis invicta* Buren (Hymenoptera: Formicidae), in Australia.. Aust J Entomol.

[37] Ramachandran S, Deshpande O, Roseman CC, Rosenberg NA, Feldman MW (2005). Support from the relationship of genetic and geographic distance in human populations for a serial founder effect originating in Africa.. Proc Natl Acad Sci USA.

[38] Brohede J, Ellegren H (1999). Microsatellite evolution: polarity of substitutions within repeats and neutrality of flanking sequences.. Proc Biol Sci.

[39] Schierup MH, Vekemans X, Charlesworth D (2000). The effect of subdivision on variation at multi-allelic loci under balancing selection.. Genet Res.

[40] Zayed A, Packer L (2005). Complementary sex determination substantially increases extinction proneness of haplodiploid populations.. Proc Natl Acad Sci USA.

[41] Zayed A (2004). Effective population size in Hymenoptera with complementary sex determination.. Heredity.

[42] Hedrick PW, Gadau J, Page REJ (2006). Genetic sex determination and extinction.. Trends Ecol Evol.

[43] Giraud T, Pedersen JS, Keller L (2002). Evolution of supercolonies: the argentine ants of Southern Europe.. Proc Natl Acad Sci USA.

[44] Tsutsui ND, Suarez AV, Grosberg RK (2003). Genetic diversity, asymmetrical aggression, and recognition in a widespread invasive species.. Proc Natl Acad Sci USA.

[45] Tsutsui ND, Suarez AV (2003). The colony structure and population biology of invasive ants.. Conserv Biol.

[46] Cane JH, Strickler K, Cane JH (2003). Exotic Nonsocial Bees (Hymenoptera: Apiformes) in North America: Ecological Implications.. For Nonnative Crops, Whence Pollinators of the Future?.

[47] Simberloff D, Drake JA, Mooney HA, di Castri F, Groves RH, Kruger FJ (1989). Which insect introductions succeed and which fail?. Biological Invasions: a Global Perspective.

[48] Knerer G (1969). Stones, cement and guards in Halictine nest architecture and defense.. Entomol News.

[49] Crawley MJ, Drake JA, Mooney HA, di Castri F, Groves RH, Kruger FJ (1989). Chance and timing in biological invasions.. Biological Invasions: a Global Perspective.

[50] Suarez AV, Holway DA, Ward PS (2005). The role of opportunity in the unintentional introduction of nonnative ants.. Proc Natl Acad Sci USA.

[51] Grevstad FS (1999). Experimental invasions using biological control introductions: the influence of release size on the chance of population establishment.. Biol Invasions.

[52] Clausen CP (1978). Introduced Parasites and Predators of Arthropod Pests And Weeds: A World Review..

[53] Hebert PND, Ratnasingham S, DeWaard JR (2003). Barcoding animal life: cytochrome *c* oxidase subunit 1 divergences among closely related species.. Proc Biol Sci.

[54] Goudet J (1995). FSTAT, version 1.2; a computer program to calculate F statistics.. Journal of Heredity.

[55] Rice WR (1989). Analysing tables of statistical tests.. Evolution.

[56] Zar JH (1999). Biostatistical Analysis..

[57] Owen RE, Packer L (1994). Estimation of the proportion of diploid males in populations of Hymenoptera.. Heredity.

[58] Danforth BN (1999). Phylogeny of the bee genus *Lasioglossum* (Hymenoptera: Halictidae) based on mitocondrial COI sequence data.. Syst Entomol.

[59] Eickwort GC, Ginsberg HS (1980). Foraging and mating behavior in Apoidea.. Annu Rev Entomol.

[60] Beveridge M, Simmons LW (2006). Panmixia: an example from Dawson's burrowing bee (*Amegilla dawsoni*) (Hymenoptera: Anthophorini).. Mol Ecol.

[61] Neumann K, Seidelmann K (2006). Microsatellites for the inference of population structures in the Red Mason bee *Osmia rufa* (Hymenoptera, Megachilidae).. Apidologie.

