# Supplementary material for: Successful Biological Invasion despite a Severe Genetic Load
Source: PLoS One. 2007 Sep 12;2(9):e868. doi: 10.1371/journal.pone.0000868 (PMC1964518; doi:10.1371/journal.pone.0000868)
Supplement: Table S1 — Sample sizes and locations for L. leucozonium (0.04 MB DOC) [file pone.0000868.s001.doc]

Table S1. Sample sizes and locations for *L. leucozonium*.

| Sample ID | Location | Lat. (°) | Long. (°)^1^ | Collection Date | Sample size^2^ |
| --- | --- | --- | --- | --- | --- |
| LL1 | Shenandoah, VA USA | 38.56 | -78.60 | 07-Jun-05 | 24 |
| LL2 | Lost River, WV USA | 38.91 | -78.86 | 09-Jun-05 | 9 |
| LL3 | Ithaca, NY USA | 42.42 | -76.52 | 20-Jun-05 | 23 |
| LL4 | Albion Hill, ON Canada | 43.93 | -79.83 | 26-Jun-05 | 24 |
| LL5 | Terracotta, ON Canada | 43.72 | -79.99 | 26-Jun-05 | 31 |
| LL6 | Joker's Hill, ON Canada | 44.01 | -79.31 | 11-Jun-05 | 24 |
| LL7 | Forks of the Credit, ON Canada | 43.49 | -80.00 | 11-Jun-05 | 21 |
| LL8 | Combermere, ON Canada | 45.21 | -77.36 | 23-Jul-05 | 19 |
| LL9 | Palmer Rapids, ON Canada | 45.17 | -77.27 | 23-Jul-05 | 21 |
| LL10 | St. Igance, NB Canada | 46.71 | -65.05 | 31-Jul-05 | 7 |
| LL11 | Avonport, NS Canada | 45.12 | -64.27 | 13-Jul-05 | 18 |
| LL12 | Northwest Cove, NS Canada | 44.32 | -64.02 | 15-Jul-05 | 15 |
| LLF | Mont Serein, Vaucluse France | 44.18 | 5.31 | 17-May-93 | 18 |

^1^ Negative values indicate west.

^2^ Number of females.
