# Supplementary figures and images for: Successful Biological Invasion despite a Severe Genetic Load
Source: PLoS One. 2007 Sep 12;2(9):e868. doi: 10.1371/journal.pone.0000868 (PMC1964518; doi:10.1371/journal.pone.0000868)

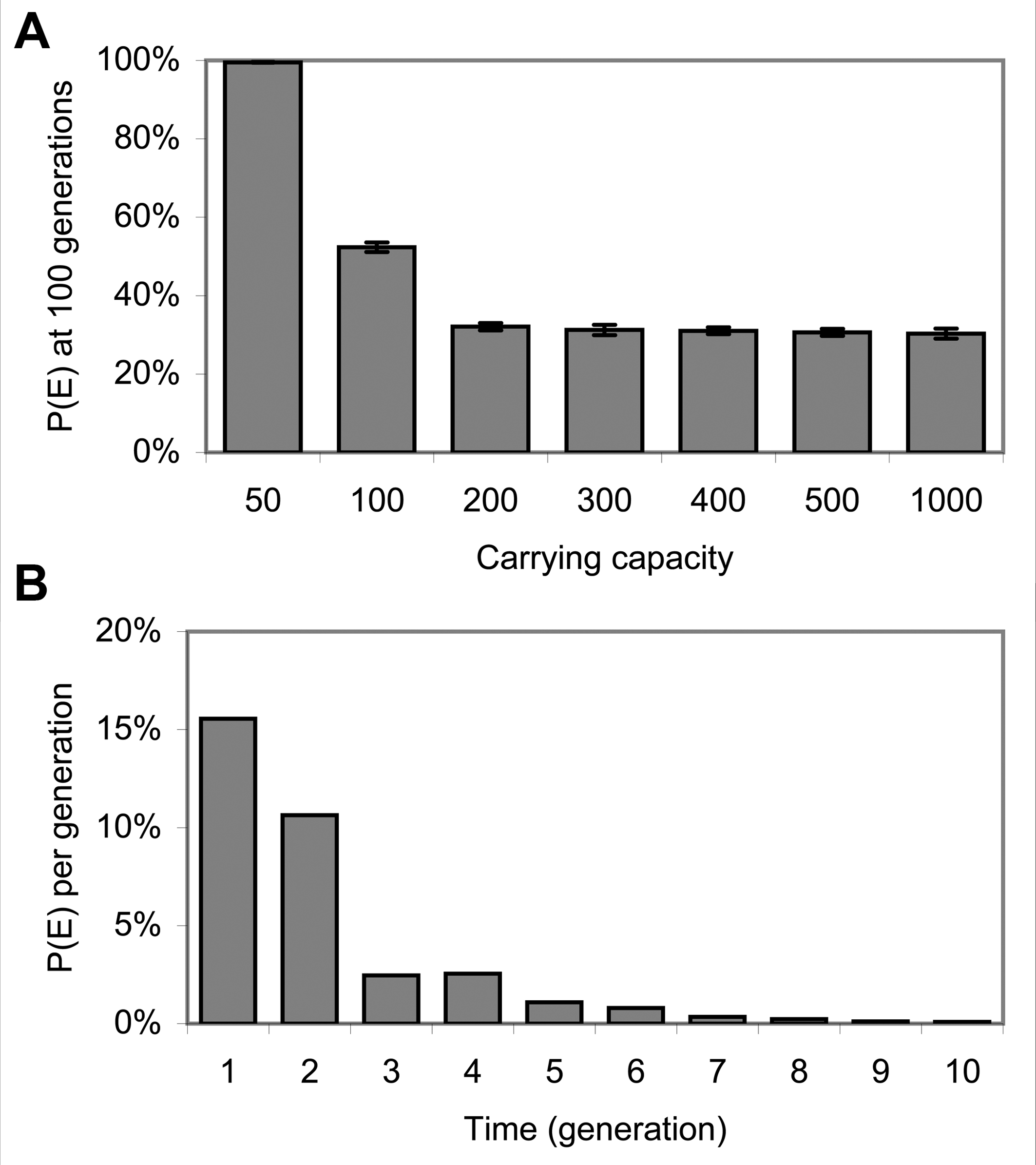

Supplement: Figure S1 — Chance and the establishment of the introduced population. (A) The probability of extinction, P(E), for a population established by one singly-mated female reached an asymptote with increasing K. Both extinction and persistence of the founder population were likely. Error bars indicate standard deviation. (B) Populations which survived the first two generations after the founding event had a negligible risk of extinction (K = 1000 bees). (5.10 MB TIF) [file pone.0000868.s002.tif]

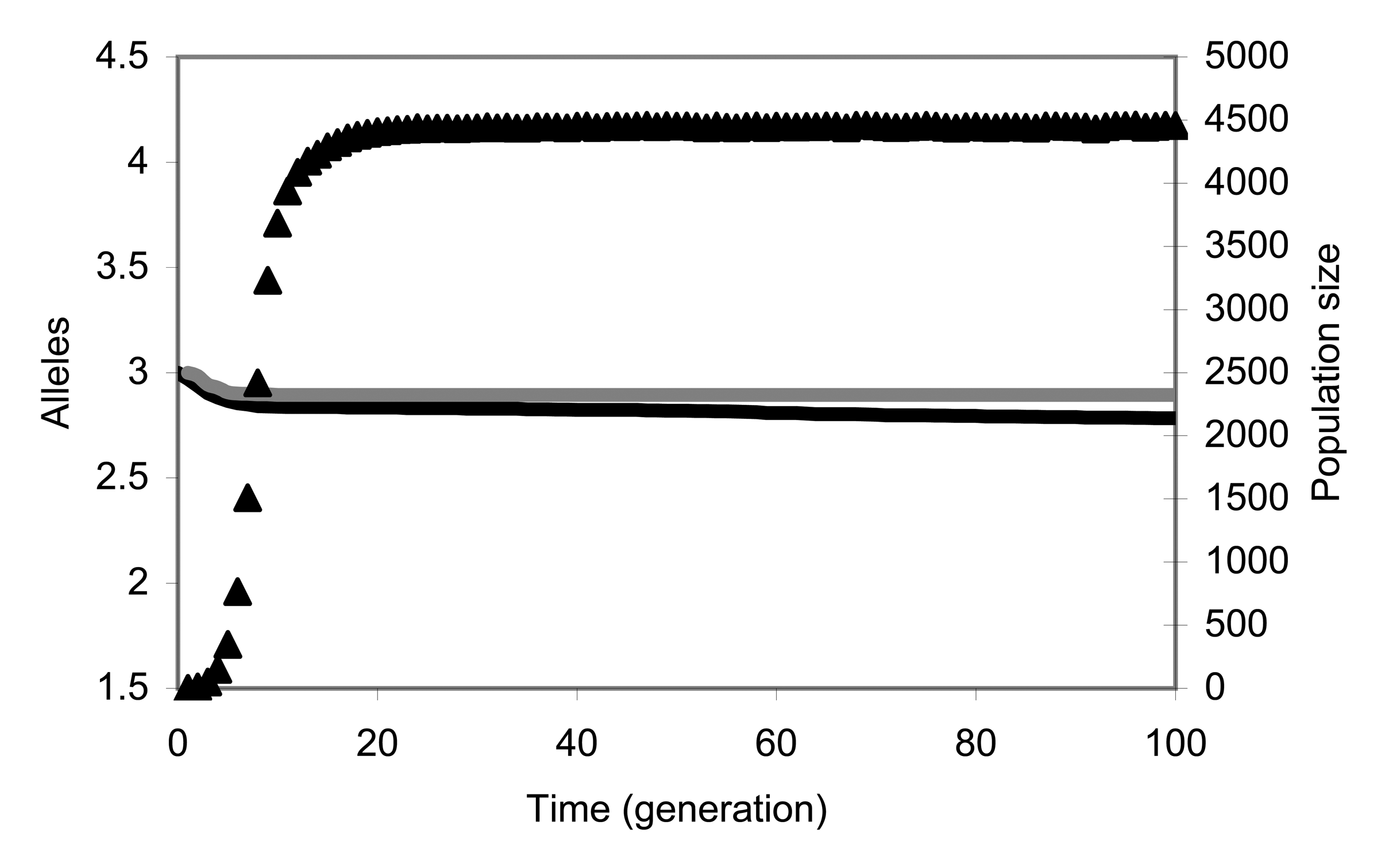

Supplement: Figure S2 — Allelic richness is maintained in a small but initially expanding founder population. The graph was generated by modeling a population with K = 5000, founded by one-singly mated female with 3 microsatellite alleles (black line) and 3 sex-determination alleles (grey line). Average allelic richness and average population size (black triangles) of extant populations are plotted on the left and right y-axis respectively. Mutation was not simulated. (3.66 MB TIF) [file pone.0000868.s003.tif]
